# Late Embryogenesis Abundant (LEA) Constitutes a Large and Diverse Family of Proteins Involved in Development and Abiotic Stress Responses in Sweet Orange (*Citrus sinensis* L. Osb.)

**DOI:** 10.1371/journal.pone.0145785

**Published:** 2015-12-23

**Authors:** Andresa Muniz Pedrosa, Cristina de Paula Santos Martins, Luana Pereira Gonçalves, Marcio Gilberto Cardoso Costa

**Affiliations:** Centro de Biotecnologia e Genética, Departamento de Ciências Biológicas, Universidade Estadual de Santa Cruz, Ilhéus, BA, Brazil; Texas Tech University, UNITED STATES

## Abstract

Late Embryogenesis Abundant (LEA) proteins are an ubiquitous group of polypeptides that were first described to accumulate during plant seed dehydration, at the later stages of embryogenesis. Since then they have also been recorded in vegetative plant tissues experiencing water limitation and in anhydrobiotic bacteria and invertebrates and, thereby, correlated with the acquisition of desiccation tolerance. This study provides the first comprehensive study about the LEA gene family in sweet orange (*Citrus sinensis* L. Osb.), the most important and widely grown fruit crop around the world. A surprisingly high number (72) of genes encoding *C*. *sinensis* LEAs (CsLEAs) were identified and classified into seven groups (LEA_1, LEA_2, LEA_3 and LEA_4, LEA_5, DEHYDRIN and SMP) based on their predicted amino acid sequences and also on their phylogenetic relationships with the complete set of *Arabidopsis thaliana* LEA proteins (AtLEAs). Approximately 60% of the CsLEAs identified in this study belongs to the unusual LEA_2 group of more hydrophobic LEA proteins, while the other LEA groups contained a relatively small number of members typically hydrophilic. A correlation between gene structure and motif composition was observed within each LEA group. Investigation of their chromosomal localizations revealed that the *CsLEAs* were non-randomly distributed across all nine chromosomes and that 33% of all *CsLEAs* are segmentally or tandemly duplicated genes. Analysis of the upstream sequences required for transcription revealed the presence of various stress-responsive *cis*-acting regulatory elements in the promoter regions of *CsLEA*s, including ABRE, DRE/CRT, MYBS and LTRE. Expression analysis using both RNA-seq data and quantitative real-time RT-PCR (qPCR) revealed that the *CsLEA* genes are widely expressed in various tissues, and that many genes containing the ABRE promoter sequence are induced by drought, salt and PEG. These results provide a useful reference for further exploration of the CsLEAs functions and applications on crop improvement.

## Introduction

Late Embryogenesis Abundant (LEA) proteins have been found to accumulate in tissues of plants exposed to stresses that include dehydration or during some stages of plant development involving water limitation, such as seed, pollen grain, shoot and root development [1]. Their name is related to the fact that the originally described proteins were observed to accumulate at high levels during the later stages of embryo development [2,3]. Since the orthodox seeds acquire the ability to withstand and tolerate desiccation at this stage of development, LEA proteins have been associated with dehydration tolerance [4,5].

LEAs are widely distributed proteins in the plant kingdom, from algae to angiosperms, and they are also found in anhydrobiotic invertebrates and in some bacterial species [1]. They are mainly composed of hydrophilic amino acids arranged in repeated sequences, forming a highly hydrophilic structure and with thermal stability [5,6]. Analyses of their amino acid sequences have separated LEA proteins into seven different groups, each containing distinctive motifs [1]. LEAs appear located in many cell types and in varying concentrations. Inside the cell they appear predominantly, but not exclusively, in the cytosol [7]. Other locations include chloroplast, mitochondria, protein and lipid bodies, plasmodesmata and nucleus [8].

The importance of LEA proteins has been inferred from their abundance and expression patterns, as well as their overexpression in transgenic plants, since the in vivo activities for most of them remain unknown. *LEA* genes have been shown to be significantly induced by abiotic stresses, such as cold, drought and salinity, and their overexpression in transgenic plants has resulted in increased tolerance to such abiotic stresses [9–14]. For these reasons, it has been postulated that there is a positive correlation between the expression of *LEAs* and abiotic stress tolerance in plants [15,16]. The observations that silencing of one or two of the three LEA_4 proteins in *Arabidopsis thaliana* is sufficient to cause water deficit susceptibility [17] and that a *DEHYDRIN* gene co-segregated with chilling tolerance during cowpea (*Vigna unguiculata* L.) seedling emergence [18] also reinforce this interpretation.

The possible functions of LEA proteins have been demonstrated by in vitro experiments, where those LEAs from groups 2, 3 and 4 were observed to prevent the inactivation of enzymes like lactate dehydrogenase (LDH) and malate dehydrogenase (MDH) upon different levels of dehydration [19–21]. Similar protective properties of LEA proteins were also observed during in vitro freeze-thaw assays and attributed to the presence of the K-segment in LEA_2 and a conserved region in LEA_4 proteins [21,22]. Some LEA and LEA-like proteins have been also shown to avoid protein aggregation in in vitro dehydration assays [19,23]. This protective activity is thought to be carried out by direct protein-protein interactions [20,24]. LEA proteins have also another interesting functional property related to its supposed function as membrane stabilizers under stress conditions [18,25–27]. Additional functions have been suggested for LEA proteins, including ion sequestration, particularly for LEA_2 and LEA_4, where histidine-containing motifs seem to bind divalent cations [28], and oxidant scavenger [28,29].

Citrus are economically important fruit crops cultivated in many tropical and subtropical areas of the world, where they are constantly exposed to a range of environmental stresses that include drought, high salinity, and extreme temperature. For this reason, efforts are required to improve their tolerance to abiotic stresses, particularly by transferring genes related to salt and drought tolerance, such as those acting in osmotic adjustment or membrane stabilization, to citrus rootstocks [30]. Although the complete set of LEA protein encoding genes has been characterized in *Arabidopsis* [31], the characterization of citrus LEAs is still rudimentary. A cDNA clone homologue to the cotton *LEA5* gene, named here as *CsLEA49*, was isolated from an ovule-derived cell suspension of ‘Shamouti’ sweet orange (*Citrus sinensis* L. Osbeck) and shown to be expressed in NaCl-treated cell suspension and leaves, as well as in seedlings exposed to drought and heat stress [32]. A cDNA clone encoding a DEHYDRIN (orthologue of *CsLEA65*) was also isolated from the epicarp of ‘Satsuma’ mandarin (*Citrus unshiu*) and observed to be expressed in different tissues and in leaves exposed to cold stress [22]. Overexpression of this gene in tobacco has enhanced the cold tolerance and inhibited the cold-induced lipid peroxidation in the transgenic plants [33]. Another cDNA encoding an orthologue DEHYDRIN was isolated from the flavedo of Fortune mandarin (*Citrus clementina* Hort. Ex Tanaka x *Citrus reticulata* Blanco) and found to be constitutively expressed in the fruit flavedo and highly induced in leaves exposed to cold and water stresses [34]. The purified protein was also observed to confer in vitro protection against freezing and dehydration inactivation for LDH and MDH enzymes [34].

The recent completion and publication of the draft genome sequences of sweet orange [35–37] now allows the identification and characterization of the complete repertoire of LEAs in citrus. Therefore, in this study we have carried out a genome-wide analysis of LEA protein encoding genes in the sweet orange genome in order to characterize their sequences, evolutionary relationships, putative functions and expression patterns in various tissues and in response to different abiotic stresses.

## Materials and Methods

### Plant material and stress treatments

For the drought stress experiment, two-year-old sweet orange [*C*. *sinensis* (L.) Osbeck var. Westin] plants grafted on the drought-tolerant citrus rootstock Rangpur lime (*C*. *limonia* Osbeck) were grown in plastic pots of 45L, containing a mixture of soil and sand (ratio 3:1), under optimal conditions (irrigated with tap water twice a week and fertilized weekly) in a greenhouse (25±4°C, 16 h of light and RH oscillating between 80 and 95%) for 90 days. After that, the pots were covered with aluminum foil to prevent water loss by evaporation, and a set of 10 plants was randomized over the experimental area and subjected to the following treatments: (i) control treatment, in which five plants were maintained at leaf predawn water potential values of -0.2 to -0.4 MPa by daily irrigation and (ii) drought treatment, in which the other five plants were exposed to a progressive soil water deficit until their leaves reach predawn water potential values of -1.5 MPa. The leaves were then harvested and immediately frozen in liquid nitrogen and stored at -80°C until use. The leaf predawn water potential was recorded on the third fully expanded mature leaf from the apex of each plant, between 2 AM and 4 AM, using a Scholander-type pressure pump (m670, Pms Instrument Co., Albany, USA).

For the salt and osmotic stress experiments, sweet orange [*C*. *sinensis* (L.) Osbeck var. Valencia] seeds were in vitro germinated as previously described [38]. Twenty-day-old seedlings of nucellar origin were selected, based on their uniformity, and then transferred to MS medium alone (control) or containing 150 mM NaCl (Merck, Darmstadt, Germany) or 25% polyethylene glycol 6000 (PEG-6000) (Merck, Darmstadt, Germany). Each treatment consisted of fifteen biological replicates. Leaves and roots were harvested 20 days after the treatments and immediately frozen in liquid nitrogen and stored at -80°C until use.

### Identification and sequence analysis of LEA protein encoding genes in the sweet orange genome

The HMM (Hidden Markov Model) profiles of the PFAM (http://pfam.sanger.ac.uk/; [39]) motifs PF03760 (LEA1), PF03168 (LEA2), PF03242 (LEA3), PF02987 (LEA4), PF00477 (LEA5), PF00257 (DEHYDRIN), and PF04927 (SMP) were used as keywords to search the sweet orange genome sequence database (http://www.phytozome.org/citrus). The sweet orange genome was also queried by the 51 *Arabidopsis* LEA protein sequences downloaded from TAIR (http://www.arabidopsis.org), using the TBLASTN tool [40]. Comparison of the sizes of the different LEA groups in other species of higher plants was performed by searching their well-annotated genomes available at Phytozome (www.phytozome.net/), using the same strategy as outlined for sweet orange.

The molecular weight (MW) and GRAVY (grand average of hydropathy) of *CsLEA* proteins were predicted by the PROTPARAM tool (http://web.expasy.org/protparam/) [41]. The subcellular localization of the proteins was predicted by the WoLF PSORT tool available at http://www.genscript.com/psort/wolf_psort.html [42]. The exon-intron structure of the sweet orange *LEA* genes was analyzed using the sweet orange gene models annotated in Phytozome.

Multiple sequence alignment of the deduced amino acid sequences of *CsLEA* proteins were performed using the default parameters of ClustalX 2 [43] and the dendrogram was constructed by the neighbor joining (NJ) method [44] and bootstrap analysis (1,000 replications) in MEGA 6 program [45]. The protein motif analysis was conducted using the program MEME/MAST (http://meme.nbcr.net/meme/) [46].

### Analysis of promoter regions and chromosomal locations of *CsLEA* genes

To identify the presence of the stress-responsive *cis*-acting regulatory elements ABRE (ABA-responsive element; ACGTG), DRE/CRT (dehydration responsive element/C-repeat; G/ACCGAC), MYBS (MYB binding site; TAACTG) and LTRE (low-temperature-responsive element; CCGAC) in their promoters, the one kb upstream region from the translation start site of the *CsLEA* genes was analyzed using the PLACE database (http://www.dna.affrc.go.jp/PLACE/signalscan.html) [47]. The physical location of each *CsLEA* was determined by confirming the starting position of all genes on each chromosome, using BLASTN searching against the local database of the *C*. *sinensis* Annotation Project (CAP; http://citrus.hzau.edu.cn/orange/). MapChart software was used to plot the gene loci on the sweet orange chromosomes [48].

### Expression analysis of *CsLEA*s

RNA-Seq data were downloaded from CAP [36] and used to generate the expression patterns of *CsLEA*s in different tissues, namely callus, flower, leaf and fruit (flesh tissue). The heatmap was generated using the Cluster 3.0 software.

Quantitative real-time RT-PCR (qPCR) analysis was used to measure the expression changes of *CsLEA*s in response to different abiotic stresses. Total RNA isolation and cDNA synthesis were performed as described previously [38]. qPCR primers were designed in order to avoid the conserved regions, by using the Primer 3 tool (http://bioinfo.ut.eeprimer3-0.4.0). *GAPC2* (Glyceraldehyde-3-phosphate dehydrogenase C2) and *UPL7* (ubiquitin protein ligase 7) were used as internal reference genes to normalize expression among the different samples [49]. Primer sequences are shown in detail in S1 Table. The qPCR reactions were run on a Stratagene Mx3005P real-time PCR System (Agilent Technologies, Santa Clara, CA, USA), following the manufacturers' instructions. The reactions were performed in triplicate, containing 10 μl (10 ng) of the cDNA sample, 10 nM of each forward and reverse primers, 12.5 μl Maxima^®^ SYBR Green/ROX qPCR Master Mix (2X) (Fermentas, Maryland, USA) and sterile Milli-Q water for a final volume of 25 μl. The thermal cycling conditions were: 10 min at 95°C and 40 cycles of 15 s at 95°C, 30s at 60°C and 30s at 72°C. Data were obtained from a pool of three biological replicates that were individually validated.

## Results and Discussion

### LEA protein encoding genes in the sweet orange genome

Existing annotation in the sweet orange genome sequence database at Phytozome and BLAST searches using the 51 amino acid sequences of the complete set of *A*. *thaliana* LEA proteins (*AtLEA*s) as query sequences have resulted in the identification of a total of 72 different *LEA* encoding protein genes in *C*. *sinensis* (*CsLEA*s), which were distributed in seven distinct groups (LEA_1, LEA_2, LEA_3, LEA_4, LEA_5, DEHYDRIN and SMP) (S2 Table). The sweet orange genome was observed to contain 4 LEA_1, 43 LEA_2, 5 LEA_3, 7 LEA_4, 3 LEA_5, 6 DEHYDRIN and 4 SMP proteins encoding genes. This number is far higher than the numbers of *LEA* genes previously reported in the genomes of rice (34) [50], *Arabidopsis* (51) [31], Chinese plum (30) [51], poplar (53) [52], tomato (27) [53] and potato (29) [54].

**Fig 1:**
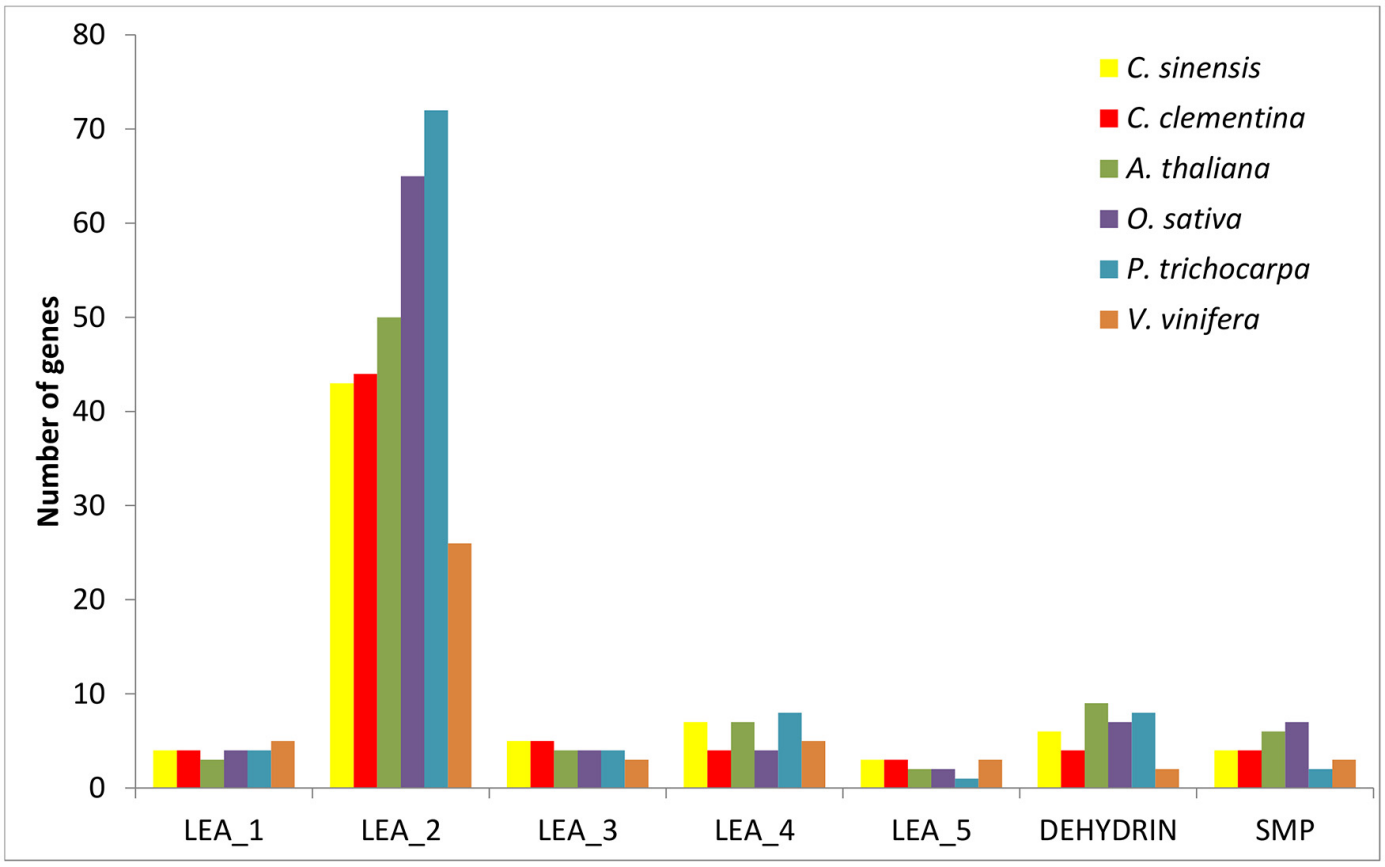
Comparison of the size of the different *LEA* gene groups in sweet orange (*Citrus sinensis*), Clementine mandarin (*Citrus clementina*), *Arabidopsis* (*Arabidopsis thaliana*), rice (*Oryza sativa*), poplar (*Populus trichocarpa*) and grapevine (*Vitis vinifera*).

In order to compare the numbers of *LEA* genes within each group among the different species of higher plants, we searched the well-annotated genomes of Clementine mandarin (*Citrus clementina*), *Arabidopsis* (*A*. *thaliana*), rice (*Oryza sativa*), poplar (*Populus trichocarpa*) and grapevine (*Vitis vinifera*) available at Phytozome, using the same strategy as outlined for sweet orange. The analysis revealed that main differences occur in the LEA_2 group (Fig 1). The abundance of LEA_2 genes is lowest in grapevine (26) and especially higher in rice (65) and poplar (72). More significantly, such a large number of LEA_2 members have not been described in the previously investigated genomes of *Arabidopsis* [31], rice [50] and poplar [52]. This result may be explained in part by the improved annotation of the higher plant genomes available at Phytozome (v10.2) and also by the fact that LEA_2 is an unusual group composed of 'atypical' LEA proteins because of their more hydrophobic character. These findings suggest that the LEA protein family in higher plants may be larger and much more complex than previously described. On the other hand, minor variations were observed in the other sweet orange LEA groups, which showed a similar number of members in the analyzed higher plant genomes. This may indicate that the LEA_2 group has evolved later in higher plants.

### Phylogenetic analysis and characteristics of *CsLEA* proteins

**Fig 2:**
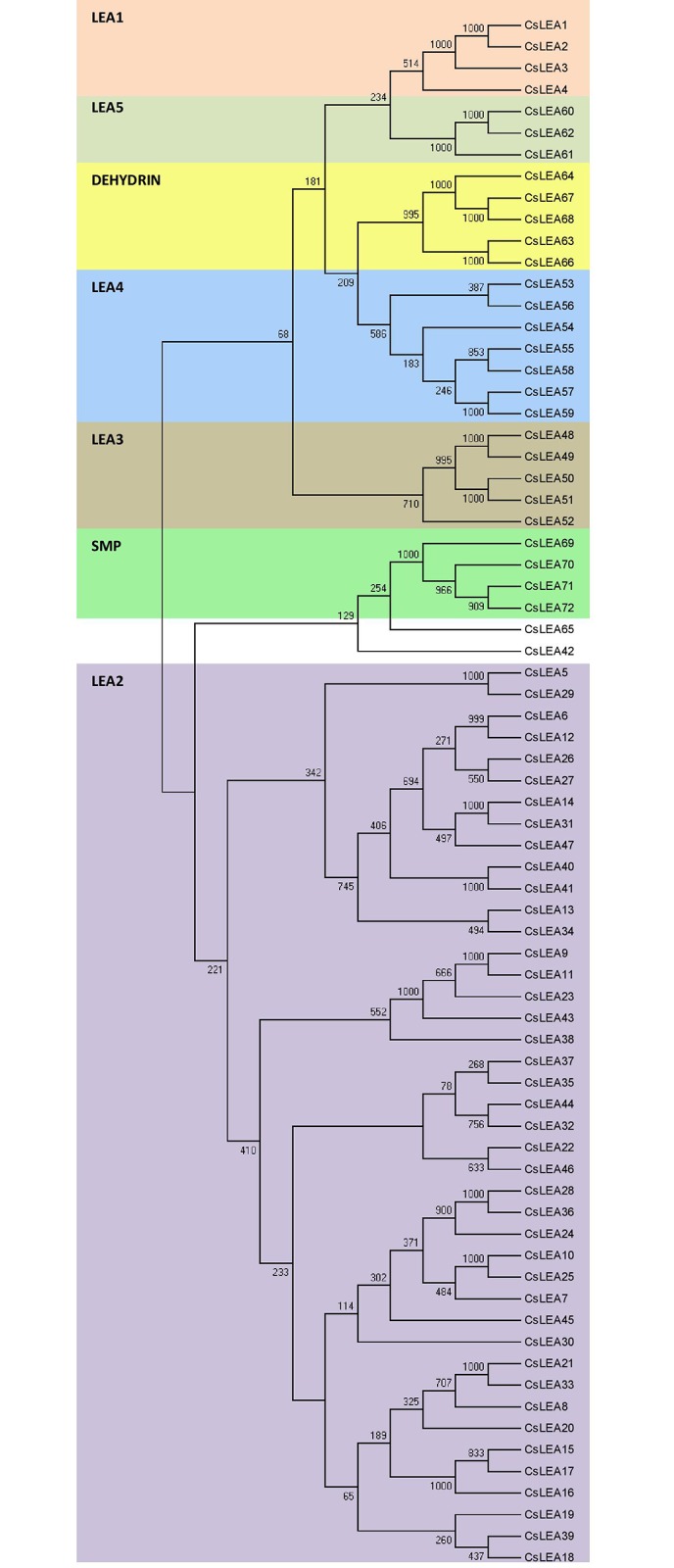
Phylogenetic comparison of the complete set of 72 different *LEA* genes (*CsLEAs*) encoded in the sweet orange genome. The different LEA groups are indicated by different colors. Sequence alignment was performed using ClustalX2 and the phylogenetic tree was generated using Bootstrap NJ tree (1,000 resamplings) method and MEGA program (v6.0.5).

All the CsLEA proteins were aligned by the NJ method in ClustalX2. The resulting dendrogram shows that they were clustered into seven groups, helping to confirm or clarify their classification as proposed in the present study (Fig 2). In the latter case, two LEA protein encoding genes (*CsLEA53* and *CsLEA54*) with no significant Pfam hits were aligned in the LEA_4 group and, hence, classified in this group. Conversely, CsLEA42 and CsLEA65 did not cluster with their respective LEA groups, despite containing the Pfam motifs that are characteristic of their groups. It may be due to the fact that CsLEA42 and CsLEA65 are, respectively, C-terminally extended and N-terminally truncated proteins compared to the other members of their respective groups.

Analysis of the physicochemical properties showed that the CsLEA proteins have a molecular weight ranging from 8.2 to 59.4 kDa, with the smaller proteins belonging to DEHYDRIN and LEA_3 groups (~8.5 kDa) and the largest proteins belonging to the LEA_4 group (59.4 kDa) (S2 Table). GRAVY values indicated that the CsLEA proteins are quite hydrophilic, except for the LEA_2 group that contains hydrophobic proteins. Similar characteristics have also been reported for LEA proteins of *Arabidopsis* [31], Chinese plum [51], poplar [52] and tomato [53], indicating that they are evolutionary conserved proteins in higher plants. The prediction of subcellular localization indicated that LEA_1 and DEHYDRIN proteins are exclusively located in the nucleus, with most members of LEA_5 and SMP and some members of LEA_2 and LEA_4 groups also targeted to this compartment (S2 Table). The majority of LEA_2 proteins are located in the cytoplasm (35.7%) or chloroplast (30.9%), with some of them also targeted to endoplasmic reticulum (RE) and mitochondrion. Most LEA_3 proteins are located in the chloroplast, while those from LEA_4 group have a more diverse localization, including chloroplast and mitochondrion (S2 Table). Thus, the CsLEA proteins can be present in all subcellular compartments, as reported for *Arabidopsis* [31] and tomato [53] LEAs; however, whether they have different functions in the different compartments, these functions need to be further determined.

**Fig 3:**
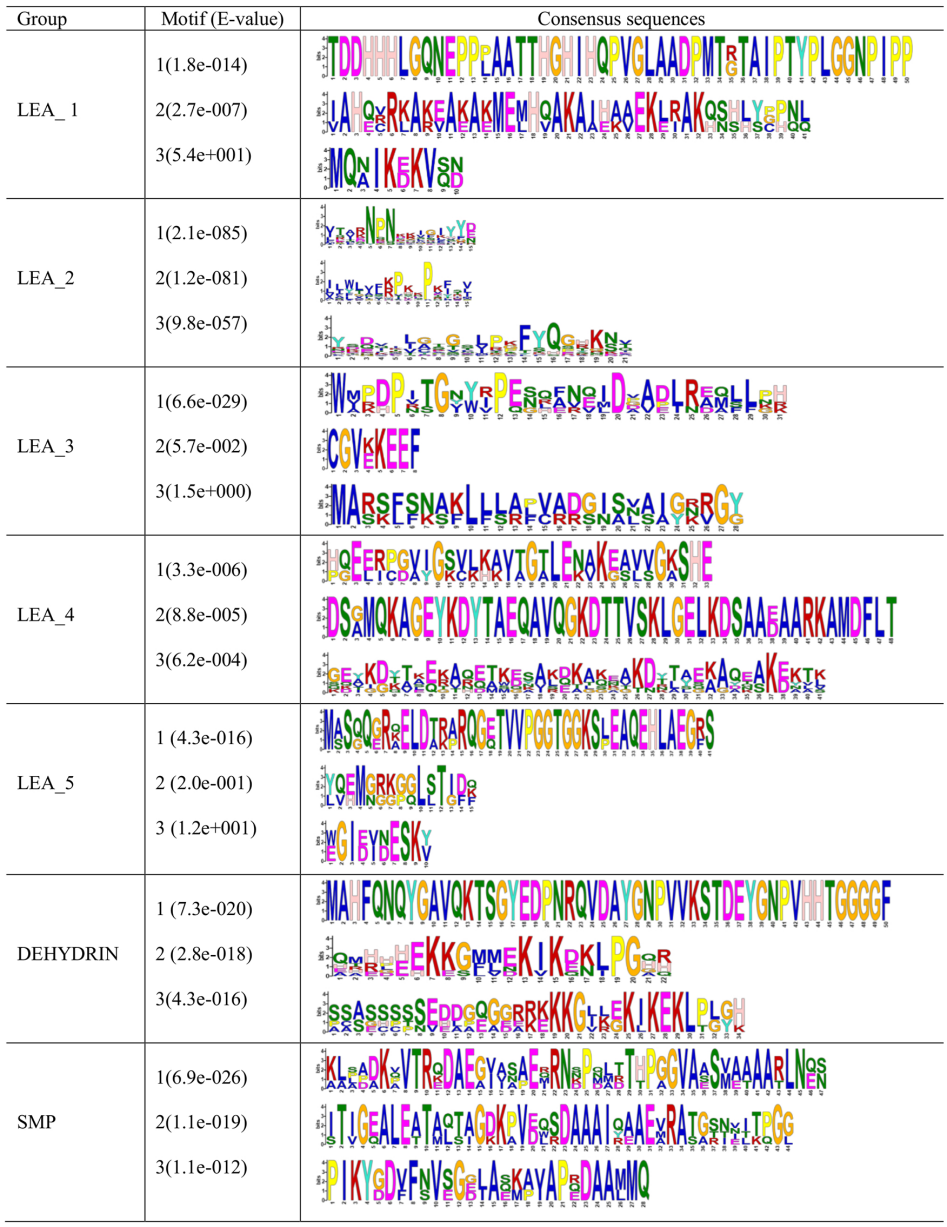
Conserved motifs in the different groups of sweet orange LEA proteins (CsLEAs). The conserved motifs were obtained using the MEME program.

Motif analysis of the predicted CsLEA proteins by the MEME program showed that members of each LEA group contain all the conserved motifs (Fig 3) that have been previously identified in other plant species, including Chinese plum [51], potato [54], tomato [53] and *Arabidopsis* [31]. For instance, an important conserved motif in the group DEHYDRIN is a repetitive 15-mer motif, EKKGIMDKIKEKLPG, called K-segment because of its richness in lysine (K) residues [1]. A particular protective role was described for this segment [20]. These results suggest that the CsLEAs are functional LEA proteins that play group-specific functions. Besides, the conserved motifs observed within each LEA group indicate that their members were likely originated from gene expansion within the groups.

### Genomic organization of *CsLEAs*

**Fig 4:**
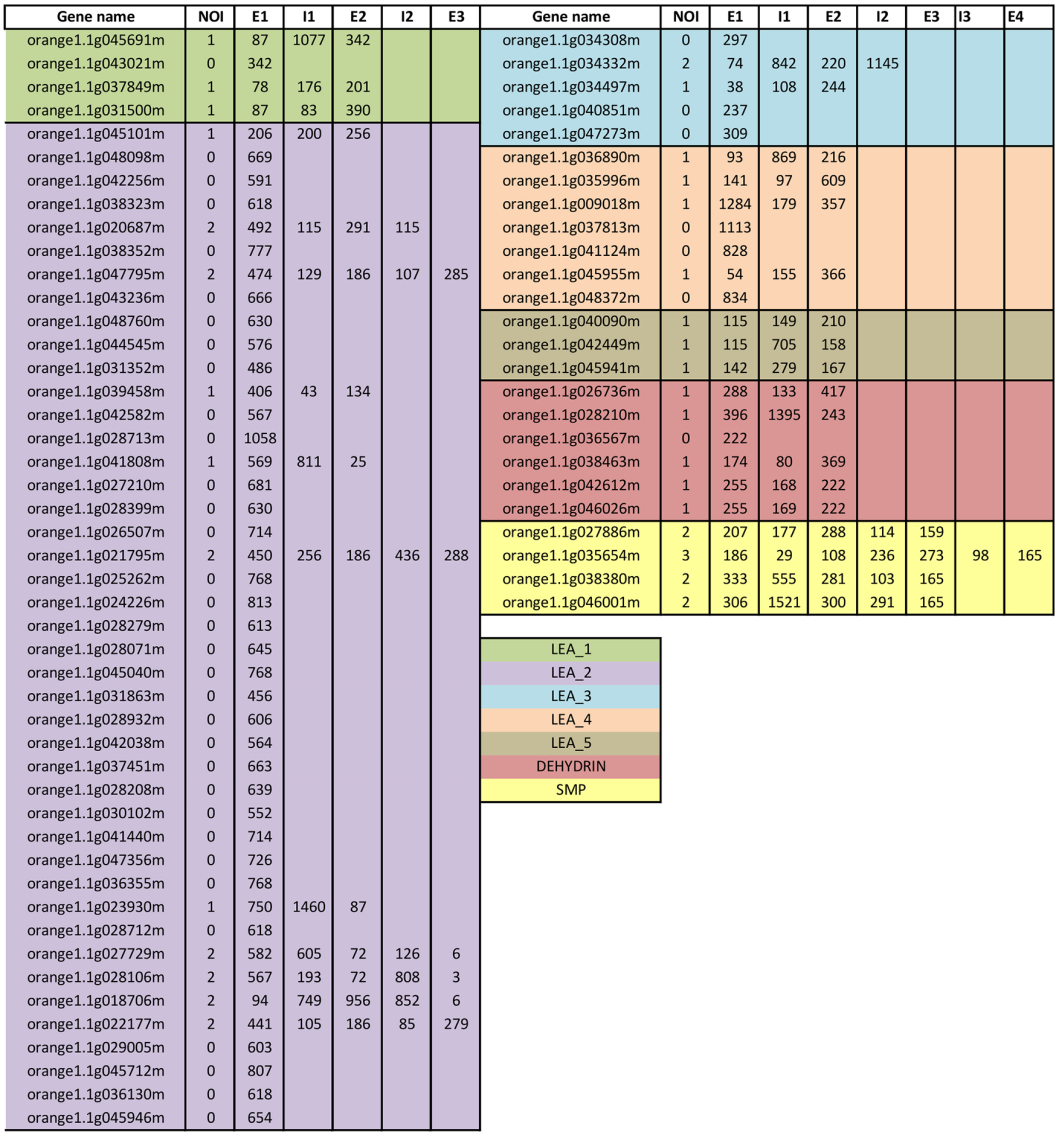
Exon-intron structure of the 72 sweet orange *LEA* genes (*CsLEAs*). NOI denotes the number of introns, E the exon and I the intron. Numbers on the E and I columns indicate, respectively, the base pair length of the exonic and intronic sequences.

Analysis of the exon-intron structure of all 72 *CsLEA* genes was carried out using the sweet orange gene models annotated in Phytozome. The number and size of the exons, but not of the introns, were usually conserved within each LEA group (Fig 4). Most *CsLEA* genes contain no or few introns, with 56% of them having no intron, 43% with one or two introns, and only one gene (*CsLEA70*) of SMP group showing three introns (Fig 4). Similar exon-intron organization were also reported in *LEA* genes of Chinese plum [51], poplar [52], tomato [53] and potato [54]. It has been argued that genes related to the stress response usually contain few introns [55]. A proposed hypothesis to explain such observation is that introns may have a deleterious effect on gene expression, since they can delay transcript production due to the various steps of splicing and processing into mature mRNA, besides the additional energetic cost caused by the increased transcript length [55].

**Fig 5:**
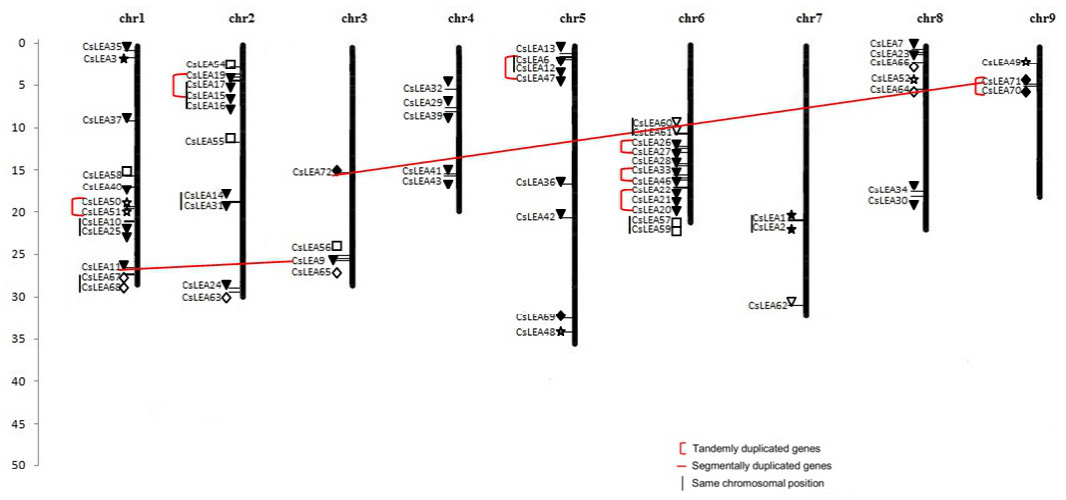
Chromosomal locations of *CsLEAs*. The chromosomal position of each *CsLEA* was mapped according to the *Citrus sinensis* Annotation Project (CAP). The *CsLEA5/29* pair of segmentally duplicated genes is not indicated in the Figure since *CsLEA5* did not map on any sweet orange chromosome (see S4 Table). The scale is in Mb. LEA_1 (closed star), LEA_2 (closed triangle), LEA_3 (open star), LEA_4 (open square), LEA_5 (open triangle), DEHYDRIN (open diamond) and SMP (closed diamond).

The positions of most *CsLEA* genes were mapped on the sweet orange chromosomes by homology searches against the full-length sweet orange genome assembly available at the *C*. *sinensis* Annotation Project (CAP) (S3 Table). Except for eight *CsLEA* genes that could not be exactly located on any chromosome (ChrUN) because of an incomplete physical map for sweet orange, all the loci were precisely mapped on the sweet orange chromosomes (Fig 5 and S4 Table). *CsLEAs* can be found on every chromosome, indicating a wide distribution of the gene family on sweet orange genome. However, the density of these loci was variable across the nine chromosomes of sweet orange. The largest number of genes was located on chromosomes 1 and 6 (12 genes each), followed by chromosome 2 (10 genes), chromosome 5 (8 genes), chromosome 8 (7 genes), chromosome 4 (5 genes), chromosomes 3 (4 genes) and chromosomes 7 and 9 (3 genes each). Members of the group LEA_1 were distributed on 2 (chr 1 and chr 7), LEA_2 on 8 (chr 1, chr 2, chr 3, chr 4, chr 5, chr 6, chr 8 and chr 9), LEA_3 on 3 (chr 1, chr 5 and chr 8), LEA_4 on 4 (chr 1, chr 2, chr 3 and chr 6), LEA_5 on 2 (chr 6 and chr 7), DEHYDRIN on 4 (chr 1, chr 2, chr 3 and chr 8) and SMP on 3 (chr 3, chr 5 and chr 9) different chromosomes. Interestingly, some closely related *CsLEA* isoforms showed significant reciprocal best BLAST hits to a single gene on CAP database and, thereby, they were mapped on the same chromosome positions (see S4 Table and Fig 5). These results might be due to the different sequencing depth and assembly quality between the sweet orange genomes available at the Phytozome and CAP databases.

Segmental duplication, tandem duplication and transposition events are the main drivers of gene family expansion. To investigate potential gene duplications in sweet orange, segmental duplications and tandem duplications were identified. We detected 12 pairs of paralogous *CsLEA* genes based on phylogenetic analysis. Among them, three pairs of paralogous genes were putative segmental duplication events, according to criteria of Gu et al. [56]: the length of aligned sequence covers >80% of the longer gene and the similarity of the aligned region is >70%. These were the LEA_2 genes *CsLEA5/29* (80.4% similarity) and *CsLEA9/11* (74.6% similarity), and the SMP genes *CsLEA71/72* (71.3% similarity). The other paralogous genes were putative tandem duplications according to the criteria of Hanada et al. [57], where tandem duplicates are genes in any gene pair, tandem 1 (T1) and tandem 2 (T2), that (1) belong to the same gene family, (2) are located within 100 kb each other, and (3) are separated by 10 or fewer nonhomologous spacer genes. These were the LEA_3 genes *CsLEA50/51* on chromosome 1, the LEA_2 genes *CsLEA15/16/17/19* on chromosome 2, *CsLEA6/12/47* on chromosome 5, *CsLEA26/27*, *CsLEA33/46* and *CsLEA20/21/22* on chromosome 6, and the SMP genes *CsLEA70/71* on chromosome 9 (S3 Table and Fig 5). Thus, these results suggest that segmental duplication and tandem duplication events have contributed to the expansion of the *LEA* gene family in sweet orange, especially in the large LEA_2 group, as proposed for the *LEA* genes of *Arabidopsis* [31], rice [50], Chinese plum [51], poplar [52], tomato [53] and potato [54].

### Stress-responsive *cis*-acting regulatory elements in the *CsLEA* promoters

**Fig 6:**
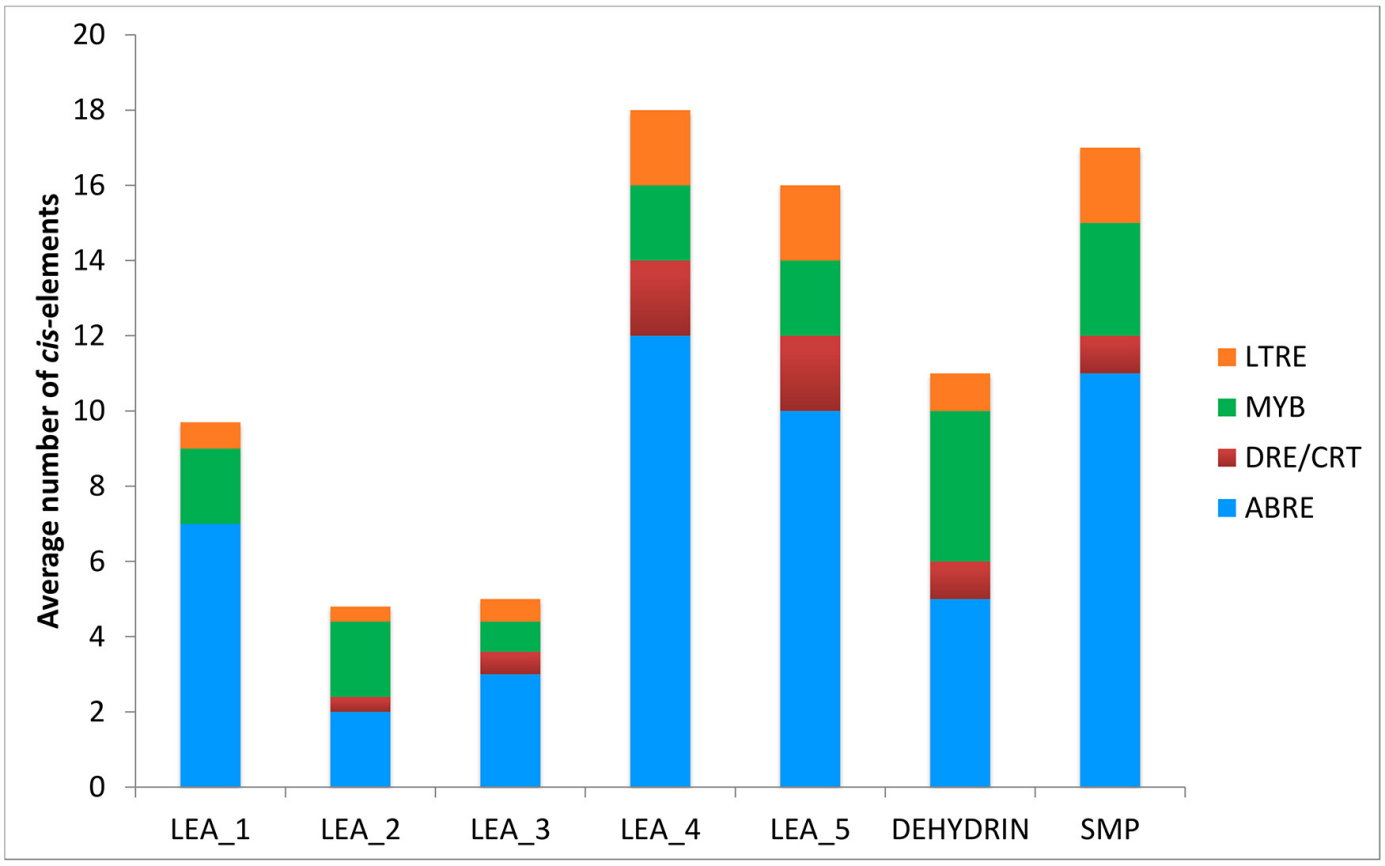
Average number of the *cis*-elements ABRE (ACGTG), DRE/CRT (G/ACCGAC), MYBS (TAACTG) and LTRE (CCGAC) in promoter region of sweet orange *LEA* genes from each LEA group. The *cis*-elements were analyzed in the 1 kb upstream promoter region of translation start site using the PLACE database.

Analysis of the promoter region of all *CsLEA* genes identified the presence of various stress responsive *cis*-acting regulatory elements, including ABRE, DRE/CRT, MYBS and LTRE. These stress-responsive elements were relatively abundant in the promoters of the *CsLEA* genes, especially ABREs (Fig 6), indicating that LEA proteins may play an important role in abiotic stress response and tolerance in sweet orange. Variations in the average number of these promoter elements were observed among the different LEA groups (Fig 6). *CsLEA* genes from LEA_4, LEA_5 and SMP groups contained the highest average number of each of the four investigated stress-responsive elements, while those from LEA_2 and LEA_3 the lowest. Similar results, with the predominance of ABRE elements, have been reported for *LEA* genes in *Arabidopsis* [31], Chinese plum [51] and tomato [53]. ABRE is the major *cis*-acting element involved in ABA signaling during seed maturation and dormancy and also in response to abiotic stresses, while DRE/CRT and LTRE are major *cis*-acting regulatory elements involved in the ABA-independent gene expression in response to dehydration (DRE/CRT) and cold (DRE/CRT and LTRE) [58]. MYBS is another well known *cis*-acting regulatory element involved in the ABA-dependent signaling pathway in response to abiotic stresses, such as drought, salt and cold [59].

### Expression patterns of *CsLEAs* in different tissues

**Fig 7:**
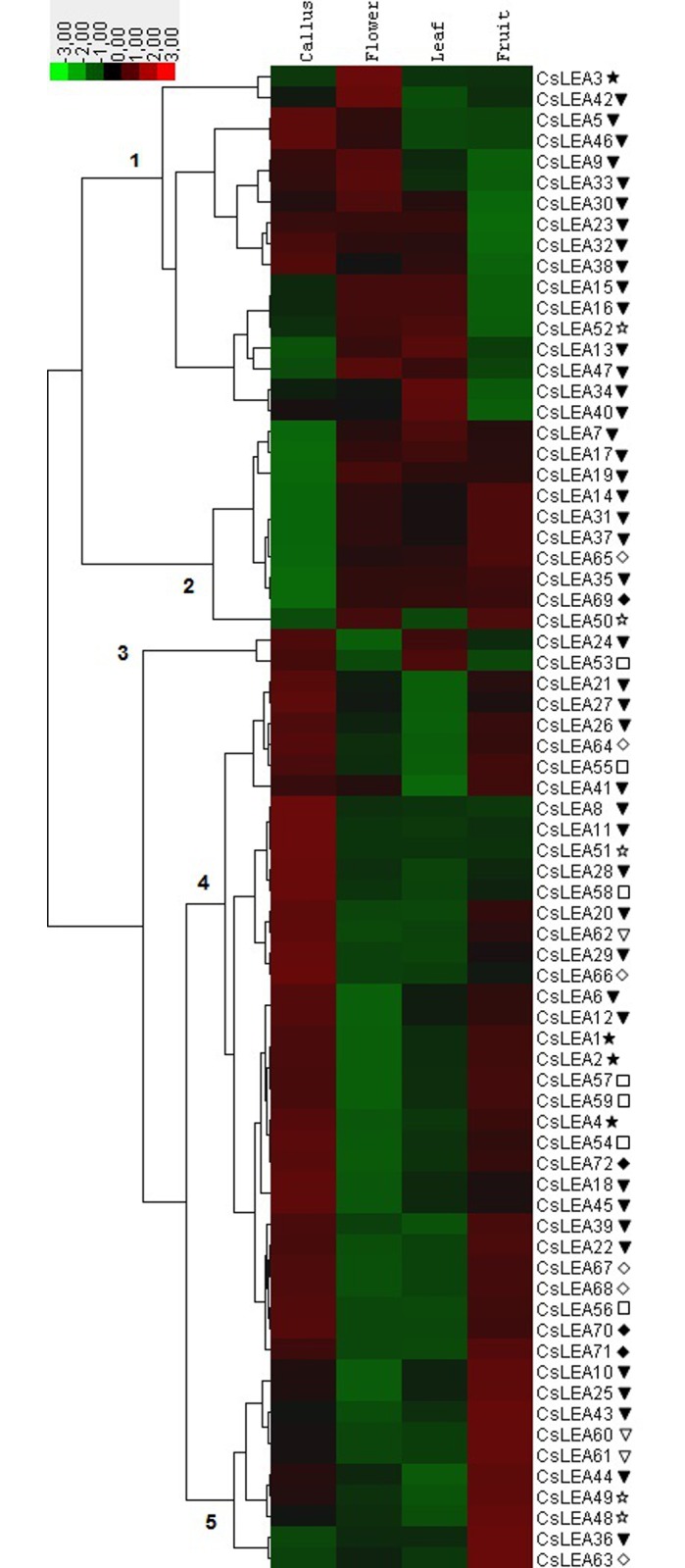
Heatmap of expression of the 72 *CsLEA* genes in different tissues of sweet orange. The heatmap was generated using Cluster 3.0 software. The color scale shown represents RPKM-normalized log_2_-transformed counts. LEA_1 (closed star), LEA_2 (closed triangle), LEA_3 (open star), LEA_4 (open square), LEA_5 (open triangle), DEHYDRIN (open diamond) and SMP (closed diamond).

In order to investigate the expression patterns of *CsLEA*s in different tissues, RNA-seq data were downloaded from CAP [36]. The heatmap generated demonstrates that all the 72 *CsLEA* genes are expressed in one or more of the major sweet orange tissues, namely callus, flower, leaf and fruit (Fig 7). The clustering revealed five main clades. *CsLEA* genes from clade 1 corresponded to family members from LEA_1, LEA_2 and LEA_3 groups displaying the highest expression in callus, flower and/or leaf tissues, while those from clade 2 included LEA_2, LEA_3 and DEHYDRIN members displaying the highest expression in flower, leaf and/or fruit. Clade 3 and clade 4 included genes from all the seven CsLEA groups with high expression, respectively, in callus and leaf or callus and fruit. Clade 5 corresponded to genes from LEA_2, LEA_3, LEA_5 and DEHYDRIN groups with high expression in fruit. Some *CsLEA*s showed preferential expression in a specific tissue, such as *CsLEA8/11/28/51/58* in callus, *CsLEA3/42* in flower and *CsLEA36/63* in fruit (Fig 7). It suggests their involvement in developmental processes that are specific to these organs. In tomato, all the *SlLEA* genes were also observed to be expressed in at least one of the ten tissues tested, with some members of the LEA_2, LEA_3 and DEHYDRIN groups showing higher expression in all the tissues [53]. On the other hand, the expression of most Chinese plum *PmLEAs* (22/30) were higher in flower, with the expression of LEA_2 and DEHYDRIN members higher than that of the other groups [51].

### Expression patterns of *CsLEAs* under abiotic stresses

**Fig 8:**
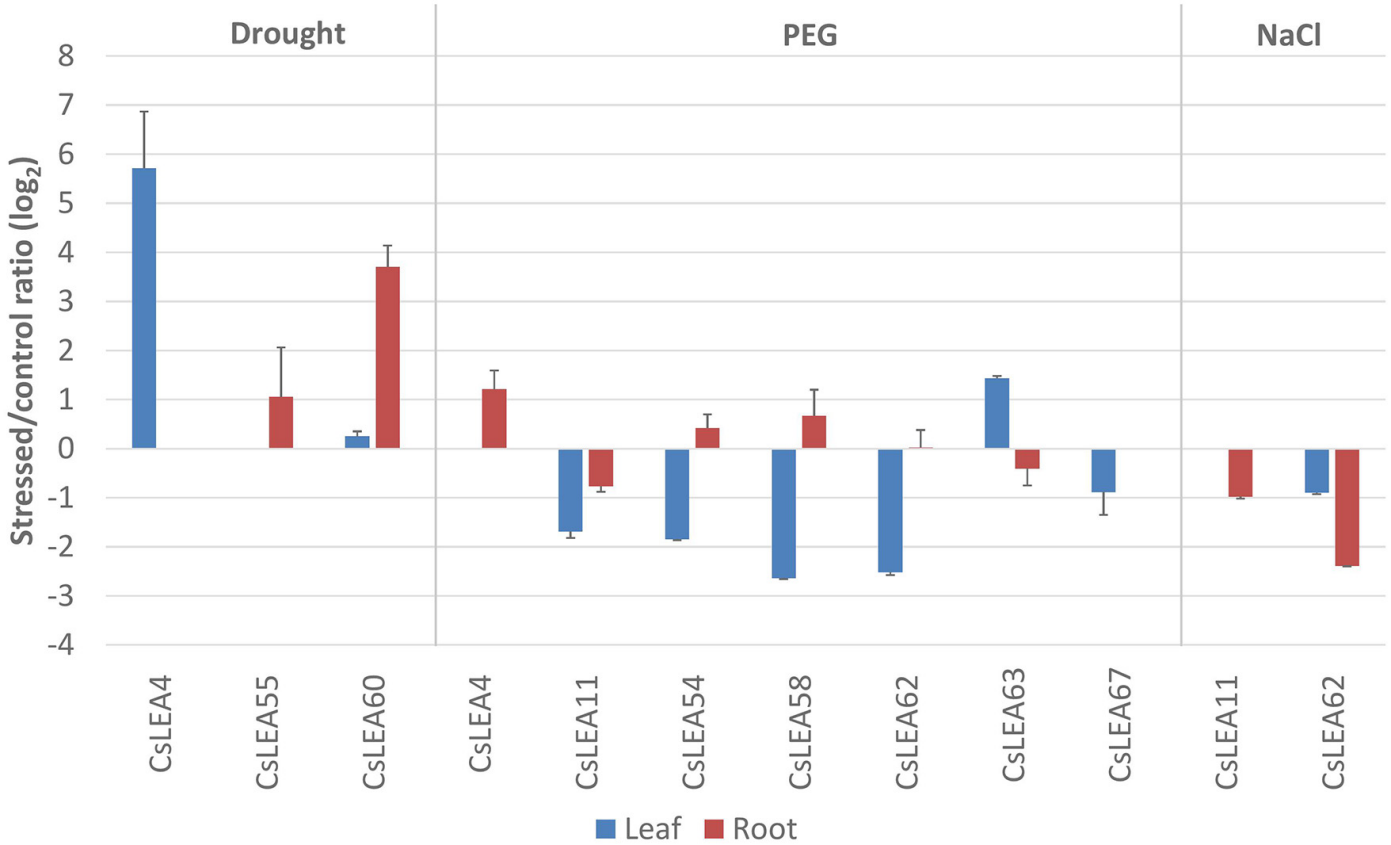
Expression profiles of the differentially expressed *CsLEA* genes in response to drought, PEG and salt (NaCl) stresses. Ratios (log_2_) of relative mRNA levels between stressed and control plants in leaves and roots, as measured by qPCR. *GAPC2* was used as an endogenous control. The bars show means ± SE from three biological replicates.

To identify *CsLEA* genes with a potential role in abiotic stress response, the expression patterns of 17 candidate genes were investigated by qPCR analysis in sweet orange plants exposed to drought, PEG and salinity. These genes were selected based on the presence of 10 or more ABRE elements in their promoter region (S5 Table). Of the 17 *CsLEA* genes analyzed, nine of them were observed to have their expression changed (log_2_ fold change of ≥1.0 or ≤-1.0 as cutoff threshold between stressed and control plants) in response to at least one stress condition and tissue analyzed (Fig 8). Analysis of expression of *CsLEA* genes under drought treatment showed an induction of mRNA expression of *CsLEA4* in leaf and *CsLEA55* and *CsLEA60* in roots (Fig 8). Osmotic stress caused by PEG treatment induced the expression of *CsLEA63* in leaf and *CsLEA4* in roots, while this treatment downregulated the expression of *CsLEA11*, *CsLEA54*, *CsLEA58*, *CsLEA62* and *CsLEA67* in leaf. On the other hand, salt treatment caused a downregulation of *CsLEA11* and *CsLEA62* in roots. Taken together, these data suggest that the differentially expressed *CsLEA* genes belonging to the LEA_1, LEA_2, LEA_4, LEA_5 and DEHYDRIN groups may play a role in the adaptation of sweet orange to the tested stress conditions. Members of these LEA groups have been found to respond to abiotic stresses like drought and salt in different plant species. For example, members of the LEA_1, LEA_3, LEA_4 and DEHYDRIN groups were observed to be induced by drought stress in *Arabidopsis*, while some members of LEA_3, LEA_4, LEA_5 and DEHYDRIN groups were salt-induced [31]. Five members of the LEA_1, LEA_2, LEA_4 and DEHYDRIN groups were upregulated by drought and salt stresses in tomato [53]. In rice, *LEA* genes of the groups LEA_2, LEA_3 and DEHYDRIN exhibited strong response to osmotic stress (PEG), salt and ABA [50].

## Conclusions

In the present study we have identified and characterized for the first time the whole repertoire of *LEA* encoding genes in the sweet orange genome. The results indicate that LEA constitute a large family of proteins in sweet orange, exhibiting a diversity of sequences, motif composition, gene structure, chromosomal locations and expression patterns. Segmental and tandem duplication events are proposed to be the main contributors to the expansion and functional diversification of the LEA gene family in sweet orange. The future efforts to elucidate their functional role, as well as to explore their potential on the genetic improvement of abiotic stress tolerance in citrus, should greatly benefit from the presented comprehensive analysis.

## Supporting Information

S1 Table Primers used in the qPCR analysis. (DOCX)

S2 Table Characteristics of genes encoding LEA proteins in sweet orange. (DOCX)

S3 Table Similarity analysis of sweet orange *LEAs* from two different databases. (DOCX)

S4 Table Chromosomal locations of *CsLEAs*. The chromosomal position of each *CsLEA* was mapped according to the *C*. *sinensis* Annotation Project (CAP).
(DOCX)

S5 Table Number of *cis*-elements ABRE (ACGTG) in the promoter regions of the selected *CsLEAs* for gene expression analysis. (DOCX)
