# Supplementary material for: Late Embryogenesis Abundant (LEA) Constitutes a Large and Diverse Family of Proteins Involved in Development and Abiotic Stress Responses in Sweet Orange (Citrus sinensis L. Osb.)
Source: PLoS One. 2015 Dec 23;10(12):e0145785. doi: 10.1371/journal.pone.0145785 (PMC4689376; doi:10.1371/journal.pone.0145785)
Supplement: S1 Table — (DOCX) [file pone.0145785.s001.docx]

**S1 Table. Primers used in the qPCR analysis.**

| **Primer name** | **Primer sequences (5´-3’)** |
| --- | --- |
| *Cs*LEA1_31500_RT-F | CCGATGGGAGTCAATACTGG |
| *Cs*LEA1_31500_RT-R | GAACCACCAGTGCCATATCC |
| *Cs*LEA2_47795_RT-F | ATGTTTTGGGCAGATTGGTC |
| *Cs*LEA2_47795_RT-R | TGCCCACATTCATCTTCTTG |
| *Cs*LEA4_37813_RT-F | CGGCTTGGGTATGCTAATGT |
| *Cs*LEA4_37813_RT-R | AAGTTTGTTTCCGCTGGTGT |
| *Cs*LEA4_41124_RT-F | CGTCAAGGCAAGAAAAATTG |
| *Cs*LEA4_41124_RT-R | TGCTGCTTGTTCGTTTTCTC |
| *Cs*LEA4_45955_RT-F | ATCCCACGATCAGAGCTACA |
| *Cs*LEA4_45955_RT-R | TCACATTCTCCATCGTCTGG |
| CsLEA4_48372_RT-F | GAACGCTGGAGAATGTCAAA |
| CsLEA4_48372_RT-R | CAGCCGATTCAGTTCCTTCT |
| *Cs*LEA4_9018_RT-F | AAGGCGGCACAATATACCAC |
| *Cs*LEA4_9018_RT-R | ATACTCAGCCGCTCCTTCAA |
| *Cs*LEA4_36890_RT-F | GCCAAAGACACTGCTCAAAA |
| *Cs*LEA4_36890_RT-R | GCTCAGCATTTTGTTTGACG |
| *Cs*LEA4_35996_RT-F | GCAGCGATGTTCATAACCAA |
| *Cs*LEA4_35996_RT-R | GACTTTGTGCGGAGTTGTGA |
| *Cs*LEA5_45941_RT-F | TGTTTCGGTTTCAGCAGTTC |
| *Cs*LEA5_45941_RT-R | TCGTCTTCGTCTGGAGTGAA |
| *Cs*LEA5_40090_RT-F | TGCATGCAGTAACAAGCTCA |
| *Cs*LEA5_40090_RT-R | CTTCCGCATTGAGACGTACA |
| *Cs*LEA5_42449_RT-F | ACGGCAACTTCATCAGCTTT |
| *Cs*LEA5_42449_RT-R | GGTGCAATCATGGTTGTTGT |
| *Cs*Deid_42612_RT-F | ATGGCACATTTTCAGAACCA |
| *Cs*Deid_42612_RT-R | TTCCCATACGCGTCTACTTG |
| CsDeid_26736_RT-F | AAGCAAACGCTCCTTGGTAA |
| CsDeid_26736_RT-R | ACTTTCCTGCGCAAATTCAT |
| *Cs*SMP_38830_RT-F | TAGCCAGCCTGAACAATGTG |
| *Cs*SMP_38830_RT-R | TGCTTCTTGCATCTCTCTGC |
| *Cs*SMP_46001_RT-F | GCAAGAACGACTCGAGATGA |
| *Cs*SMP_46001_RT-R | TTCCGTGTCACTTGCTTGTC |
| *Cs*SMP_27886_RT-F | TCTTGGACGCGATCAATCTA |
| *Cs*SMP_27886_RT-R | TCTCTCTGATCCACCGCTTT |
