# Supplementary material for: Late Embryogenesis Abundant (LEA) Constitutes a Large and Diverse Family of Proteins Involved in Development and Abiotic Stress Responses in Sweet Orange (Citrus sinensis L. Osb.)
Source: PLoS One. 2015 Dec 23;10(12):e0145785. doi: 10.1371/journal.pone.0145785 (PMC4689376; doi:10.1371/journal.pone.0145785)
Supplement: S2 Table — (DOCX) [file pone.0145785.s002.docx]

**S2 Table. Characteristics of genes encoding LEA proteins in sweet orange*.***

| **Gene name** | **Locus** | **Pfam family** | **LEA group** | **Polypeptide length**  **(MW)** | **GRAVY** | **Predicted** **subcellular** **localization** |
| --- | --- | --- | --- | --- | --- | --- |
| *CsLEA1* | orange1.1g045691m | PF03760 | LEA_1 | 142  (15.4 kDa) | -0.830 | Nucleus |
| *CsLEA2* | orange1.1g043021m | PF03760 | LEA_1 | 113  (12.3 kDa) | -0.835 | Nucleus |
| *CsLEA3* | orange1.1g037849m | PF03760 | LEA_1 | 92  (10.3 kDa) | -0.958 | Nucleus |
| *CsLEA4* | orange1.1g031500m | PF03760 | LEA_1 | 158  (16.2 kDa) | -0.941 | Nucleus |
| *CsLEA5* | orange1.1g045101m | PF03168 | LEA_2 | 153  (16.8 kDa) | -0.010 | Cytoplasm |
| *CsLEA6* | orange1.1g048098m | PF03168 | LEA_2 | 222  (24.6 kDa) | 0.073 | Cytoplasm |
| *CsLEA7* | orange1.1g042256m | PF03168 | LEA_2 | 196  (22.1 kDa) | -0.002 | Plasma membrane |
| *CsLEA8* | orange1.1g038323m | PF03168 | LEA_2 | 205  (22.2 kDa) | 0.232 | Chloroplast |
| *CsLEA9* | orange1.1g020687m | PF03168 | LEA_2 | 322  (35.4 kDa) | -0.415 | Endoplasmic reticulum |
| *CsLEA10* | orange1.1g038352m | PF03168 | LEA_2 | 258  (28.4 kDa) | -0.153 | Chloroplast |
| *CsLEA11* | orange1.1g047795m | PF03168 | LEA_2 | 314  (34.9 kDa) | -0.318 | Endoplasmic reticulum |
| *CsLEA12* | orange1.1g043236m | PF03168 | LEA_2 | 222  (24.6 kDa) | -0.105 | Cytoplasm |
| *CsLEA13* | orange1.1g048760m | PF03168 | LEA_2 | 209  (22.4 kDa) | 0.293 | Chloroplast/Mitochondrion |
| *CsLEA14* | orange1.1g044545m | PF03168 | LEA_2 | 191  (20.8 kDa) | 0.430 | Plasma membrane |
| *CsLEA15* | orange1.1g031352m | PF03168 | LEA_2 | 161  (18.2 kDa) | -0.013 | Chloroplast |
| *CsLEA16* | orange1.1g039458m | PF03168 | LEA_2 | 179  (20.8 kDa) | -0.240 | Extracellular |
| *CsLEA17* | orange1.1g042582m | PF03168 | LEA_2 | 189  (21.7 kDa) | -0.193 | Mitochondrion |
| *CsLEA18* | orange1.1g028713m | PF03168 | LEA_2 | 205  (23.4 kDa) | -0.040 | Cytoplasm |
| *CsLEA19* | orange1.1g041808m | PF03168 | LEA_2 | 197  (21.9 kDa) | -0.281 | Chloroplast |
| *CsLEA20* | orange1.1g027210m | PF03168 | LEA_2 | 226  (25.3 kDa) | -0.180 | Chloroplast |
| *CsLEA21* | orange1.1g028399m | PF03168 | LEA_2 | 209  (23.8 kDa) | 0.052 | Cytoplasm |
| *CsLEA22* | orange1.1g026507m | PF03168 | LEA_2 | 237  (26.5 kDa) | -0.068 | Cytoplasm |
| *CsLEA23* | orange1.1g021795m | PF03168 | LEA_2 | 307  (34.5 kDa) | -0.379 | Chloroplast |
| *CsLEA24* | orange1.1g025262m | PF03168 | LEA_2 | 255  (28.3 kDa) | -0.166 | Cytoplasm |
| *CsLEA25* | orange1.1g024226m | PF03168 | LEA_2 | 270  (29.2 kDa) | -0.215 | Chloroplast |
| *CsLEA26* | orange1.1g028279m | PF03168 | LEA_2 | 211  (23.5 kDa) | 0.265 | Nucleus |
| *CsLEA27* | orange1.1g028071m | PF03168 | LEA_2 | 214  (22.7 kDa) | 0.161 | Plasma membrane |
| *CsLEA28* | orange1.1g045040m | PF03168 | LEA_2 | 255  (28.9 kDa) | -0.338 | Cytoplasm |
| *CsLEA29* | orange1.1g031863m | PF03168 | LEA_2 | 151  (16.5 kDa) | -0.072 | Cytoplasm |
| *CsLEA30* | orange1.1g028932m | PF03168 | LEA_2 | 201  (22.3 kDa) | 0.135 | Chloroplast |
| *CsLEA31* | orange1.1g042038m | PF03168 | LEA_2 | 187  (20.4 kDa) | 0.199 | Extracellular |
| *CsLEA32* | orange1.1g037451m | PF03168 | LEA_2 | 220  (25.5 kDa) | -0.090 | Nucleus |
| *CsLEA33* | orange1.1g028208m | PF03168 | LEA_2 | 212  (23.3 kDa) | 0.144 | Cytoplasm |
| *CsLEA34* | orange1.1g030102m | PF03168 | LEA_2 | 183  (20.1 kDa) | 0.178 | Extracellular |
| *CsLEA35* | orange1.1g041440m | PF03168 | LEA_2 | 237  (26.8 kDa) | -0.348 | Cytoplasm |
| *CsLEA36* | orange1.1g047356m | PF03168 | LEA_2 | 241  (26.9 kDa) | -0.121 | Cytoplasm |
| *CsLEA37* | orange1.1g036355m | PF03168 | LEA_2 | 255  (28.7 kDa) | -0.307 | Chloroplast |
| *CsLEA38* | orange1.1g023930m | PF03168 | LEA_2 | 275  (30.0 kDa) | -0.174 | Endoplasmic reticulum |
| *CsLEA39* | orange1.1g028712m | PF03168 | LEA_2 | 205  (22.8 kDa) | -0.056 | Plasma membrane |
| *CsLEA40* | orange1.1g027729m | PF03168 | LEA_2 | 219  (24.8 kDa) | -0.043 | Cytoplasm |
| *CsLEA41* | orange1.1g028106m | PF03168 | LEA_2 | 213  (24.0 kDa) | 0.050 | Cytoplasm |
| *CsLEA42* | orange1.1g018706m | PF03168 | LEA_2 | 351  (38.9 kDa) | -0.342 | Chloroplast |
| *CsLEA43* | orange1.1g022177m | PF03168 | LEA_2 | 301  (33.7 kDa) | -0.490 | Chloroplast |
| *CsLEA44* | orange1.1g029005m | PF03168 | LEA_2 | 200  (22.4 kDa) | 0.203 | Chloroplast |
| *CsLEA45* | orange1.1g045712m | PF03168 | LEA_2 | 268  (30.3 kDa) | -0.115 | Nucleus |
| *CsLEA46* | orange1.1g036130m | PF03168 | LEA_2 | 205  (23.5 kDa) | 0.097 | Vacuole |
| *CsLEA47* | orange1.1g045946m | PF03168 | LEA_2 | 214  (23.6 kDa) | -0.215 | Cytoplasm |
| *CsLEA48* | orange1.1g034308m | PF03242 | LEA_3 | 98  (10.3 kDa) | -0.281 | Chloroplast |
| *CsLEA49* | orange1.1g034332m | PF03242 | LEA_3 | 97  (10.5 kDa) | -0.221 | Mitochondrion |
| *CsLEA50* | orange1.1g034497m | PF03242 | LEA_3 | 93  (10.4 kDa) | -0.560 | Chloroplast |
| *CsLEA51* | orange1.1g040851m | PF03242 | LEA_3 | 78  (8.7 kDa) | -0.603 | Chloroplast |
| *CsLEA52* | orange1.1g047273m | PF03242 | LEA_3 | 102  (11.6 kDa) | -1.090 | Cytoplasm |
| *CsLEA53* | orange1.1g036890m | - | LEA_4 | 102  (11.2 kDa) | -0.873 | Chloroplast |
| *CsLEA54* | orange1.1g035996m | - | LEA_4 | 249  (27.8 kDa) | -1.391 | Chloroplast |
| *CsLEA55* | orange1.1g009018m | PF02987 | LEA_4 | 546  (59.4 kDa) | -1.159 | Nucleus |
| *CsLEA56* | orange1.1g037813m | PF02987 | LEA_4 | 371  (40.0 kDa) | -0,512 | Extracellular |
| *CsLEA57* | orange1.1g041124m | PF02987 | LEA_4 | 276  (30.2 kDa) | -1,250 | Mitochondrion |
| *CsLEA58* | orange1.1g045955m | PF02987 | LEA_4 | 139  (15.0 kDa) | -1.429 | Mitochondrion |
| *CsLEA59* | orange1.1g048372m | PF02987 | LEA_4 | 278  (30.3 kDa) | -1.231 | Nucleus |
| *CsLEA60* | orange1.1g040090m | PF00477 | LEA_5 | 112  (12.1 kDa) | -1.344 | Cytoplasm |
| *CsLEA61* | orange1.1g042449m | PF00477 | LEA_5 | 90  (9.6 kDa) | -0.479 | Nucleus |
| *CsLEA62* | orange1.1g045941m | PF00477 | LEA_5 | 102  (10.8 Da) | -1.217 | Nucleus |
| *CsLEA63* | orange1.1g026736m | PF00257 | DEHYDRIN | 234  (26.7 kDa) | -1.654 | Nucleus |
| *CsLEA64* | orange1.1g028210m | PF00257 | DEHYDRIN | 212  (22.4 kDa) | -0.960 | Nucleus |
| *CsLEA65* | orange1.1g036567m | PF00257 | DEHYDRIN | 73  (8.2 kDa) | -1.540 | Nucleus |
| *CsLEA66* | orange1.1g038463m | PF00257 | DEHYDRIN | 180  (20.1 kDa) | -1.382 | Nucleus |
| *CsLEA67* | orange1.1g042612m | PF00257 | DEHYDRIN | 158  (16.6 kDa) | -1.145 | Nucleus |
| *CsLEA68* | orange1.1g046026m | PF00257 | DEHYDRIN | 158  (16.6 kDa) | -1.141 | Nucleus |
| *CsLEA69* | orange1.1g027886m | PF04927 | SMP | 217  (22.3 kDa) | -0.439 | Nucleus |
| *CsLEA70* | orange1.1g035654m | PF04927 | SMP | 243  (25.0 kDa) | -0.328 | Nucleus |
| *CsLEA71* | orange1.1g038380m | PF04927 | SMP | 261  (26.6 kDa) | -0,457 | Cytoplasm |
| *CsLEA72* | orange1.1g046001m | PF04927 | SMP | 256  (26.4 kDa) | -0,320 | Chloroplast |
