# Supplementary material for: Late Embryogenesis Abundant (LEA) Constitutes a Large and Diverse Family of Proteins Involved in Development and Abiotic Stress Responses in Sweet Orange (Citrus sinensis L. Osb.)
Source: PLoS One. 2015 Dec 23;10(12):e0145785. doi: 10.1371/journal.pone.0145785 (PMC4689376; doi:10.1371/journal.pone.0145785)
Supplement: S3 Table — (DOCX) [file pone.0145785.s003.docx]

**S3 Table. Similarity analysis of sweet orange *LEAs* from two different databases.**

| Gene name | ID (Phytozome) | ID (Orange Genome Annotation Project) | Identity (%) | E-value | Score |
| --- | --- | --- | --- | --- | --- |
| *CsLEA1* | orange1.1g045691 | Cs7g23030 | 100 | 0 | 951 |
| *CsLEA2* | orange1.1g043021 | Cs7g23030 | 100 | 3e-176 | 618 |
| *CsLEA3* | orange1.1g037849 | Cs1g02640 | 100 | 0 | 822 |
| *CsLEA4* | orange1.1g031500 | orange1.1t02243 | 98 | 0 | 1383 |
| *CsLEA5* | orange1.1g045101 | orange1.1t00426 | 100 | 0 | 1195 |
| *CsLEA6* | orange1.1g048098 | Cs5g02920 | 75 | 0.33 | 37 |
| *CsLEA7* | orange1.1g042256 | Cs8g01590 | 93 | 0.08 | 39 |
| *CsLEA8* | orange1.1g038323 | orange1.1t00573 | 100 | 0 | 1116 |
| *CsLEA9* | orange1.1g020687 | Cs3g23380 | 100 | 0 | 2261 |
| *CsLEA10* | orange1.1g038352 | Cs1g18130 | 70 | 1e-19 | 99 |
| *CsLEA11* | orange1.1g047795 | Cs1g22530 | 100 | 0 | 2099 |
| *CsLEA12* | orange1.1g043236 | Cs5g02920 | 100 | 0 | 1202 |
| *CsLEA13* | orange1.1g048760 | Cs5g02130 | 100 | 0 | 1128 |
| *CsLEA14* | orange1.1g044545 | Cs2g21710 | 65 | 2e-08 | 61 |
| *CsLEA15* | orange1.1g031352 | Cs2g07520 | 69 | 7e-53 | 209 |
| *CsLEA16* | orange1.1g039458 | Cs2g07520 | 100 | 0 | 1044 |
| *CsLEA17* | orange1.1g042582 | Cs2g07510 | 100 | 0 | 1024 |
| *CsLEA18* | orange1.1g028713 | orange1.1t00850 | 96 | 0.15 | 39 |
| *CsLEA19* | orange1.1g041808 | Cs2g07500 | 100 | 0 | 1193 |
| *CsLEA20* | orange1.1g027210 | Cs6g13820 | 99 | 0 | 1722 |
| *CsLEA21* | orange1.1g028399 | Cs6g13810 | 99 | 0 | 1918 |
| *CsLEA22* | orange1.1g026507 | Cs6g13800 | 99 | 0 | 1610 |
| *CsLEA23* | orange1.1g021795 | Cs8g01980 | 99 | 0 | 3045 |
| *CsLEA24* | orange1.1g025262 | Cs2g29120 | 99 | 0 | 1819 |
| *CsLEA25* | orange1.1g024226 | Cs1g18130 | 99 | 0 | 2429 |
| *CsLEA26* | orange1.1g028279 | Cs6g10540 | 99 | 0 | 1567 |
| *CsLEA27* | orange1.1g028071 | Cs6g10550 | 100 | 0 | 1552 |
| *CsLEA28* | orange1.1g045040 | Cs6g11040 | 99 | 0 | 1354 |
| *CsLEA29* | orange1.1g031863 | Cs4g10930 | 100 | 0 | 818 |
| *CsLEA30* | orange1.1g028932 | Cs8g15430 | 100 | 0 | 1182 |
| *CsLEA31* | orange1.1g042038 | Cs2g21710 | 100 | 0 | 1013 |
| *CsLEA32* | orange1.1g037451 | Cs4g08120 | 99 | 0 | 1170 |
| *CsLEA33* | orange1.1g028208 | Cs6g12560 | 100 | 0 | 1154 |
| *CsLEA34* | orange1.1g030102 | Cs8g14650 | 99 | 0 | 1329 |
| *CsLEA35* | orange1.1g041440 | Cs1g01340 | 100 | 0 | 1283 |
| *CsLEA36* | orange1.1g036355 | Cs5g17030 | 100 | 0 | 1381 |
| *CsLEA37* | orange1.1g036355 | Cs1g08730 | 100 | 0 | 1381 |
| *CsLEA38* | orange1.1g023930 | orange1.1t01171 | 99 | 0 | 4676 |
| *CsLEA39* | orange1.1g028712 | Cs4g11220 | 100 | 0 | 1408 |
| *CsLEA40* | orange1.1g027729 | Cs1g14360 | 99 | 0 | 2941 |
| *CsLEA41* | orange1.1g028106 | Cs4g15720 | 100 | 0 | 3714 |
| *CsLEA42* | orange1.1g018706 | Cs5g20510 | 98 | 0 | 5310 |
| *CsLEA43* | orange1.1g022177 | Cs4g16000 | 100 | 0 | 2393 |
| *CsLEA44* | orange1.1g029005 | orange1.1t03593 | 100 | 0 | 1814 |
| *CsLEA45* | orange1.1g045712 | orange1.1t00850 | 100 | 0 | 1442 |
| *CsLEA46* | orange1.1g036130 | Cs6g12590 | 100 | 0 | 1116 |
| *CsLEA47* | orange1.1g045946 | Cs5g02930 | 99 | 0 | 1137 |
| *CsLEA48* | orange1.1g034308 | Cs5g33290 | 99 | 0 | 2156 |
| *CsLEA49* | orange1.1g034332 | Cs9g04210 | 90 | 0 | 1619 |
| *CsLEA50* | orange1.1g034497 | Cs1g16630 | 97 | 0 | 665 |
| *CsLEA51* | orange1.1g040851 | Cs1g16640 | 95 | 2e-105 | 382 |
| *CsLEA52* | orange1.1g047273 | Cs8g04470 | 96 | 4e-142 | 504 |
| *CsLEA53* | orange1.1g036890 | orange1.1t01056 | 97 | 0 | 2044 |
| *CsLEA54* | orange1.1g035996 | Cs2g05770 | 100 | 0 | 1550 |
| *CsLEA55* | orange1.1g009018 | Cs2g15720 | 99 | 0 | 3186 |
| *CsLEA56* | orange1.1g037813 | Cs3g22950 | 100 | 0 | 2529 |
| *CsLEA57* | orange1.1g041124 | Cs6g15810 | 96 | 0 | 2024 |
| *CsLEA58* | orange1.1g045955 | Cs1g13170 | 100 | 0 | 824 |
| *CsLEA59* | orange1.1g048372 | Cs6g15810 | 100 | 0 | 2276 |
| *CsLEA60* | orange1.1g040090 | Cs6g08740 | 84 | 3e-38 | 348 |
| *CsLEA61* | orange1.1g042449 | Cs6g08740 | 81 | 9e-23 | 109 |
| *CsLEA62* | orange1.1g045941 | Cs7g30920 | 100 | 0 | 1062 |
| *CsLEA63* | orange1.1g026736 | Cs3g26970 | 100 | 0 | 3736 |
| *CsLEA64* | orange1.1g028210 | Cs8g07520 | 100 | 0 | 2673 |
| *CsLEA65* | orange1.1g036567 | Cs3g24820 | 91 | 2e-87 | 322 |
| *CsLEA66* | orange1.1g038463 | Cs8g01990 | 97 | 0 | 1149 |
| *CsLEA67* | orange1.1g042612 | Cs1g24590 | 98 | 0 | 1112 |
| *CsLEA68* | orange1.1g046026 | Cs1g24590 | 100 | 0 | 1166 |
| *CsLEA69* | orange1.1g027886 | Cs5g31080 | 100 | 0 | 1925 |
| *CsLEA70* | orange1.1g035654 | Cs9g07030 | 99 | 0 | 1940 |
| *CsLEA71* | orange1.1g038380 | Cs9g07010 | 100 | 0 | 2781 |
| *CsLEA72* | orange1.1g046001 | Cs3g11550 | 92 | 9 | 3451 |
