# Supplementary material for: Late Embryogenesis Abundant (LEA) Constitutes a Large and Diverse Family of Proteins Involved in Development and Abiotic Stress Responses in Sweet Orange (Citrus sinensis L. Osb.)
Source: PLoS One. 2015 Dec 23;10(12):e0145785. doi: 10.1371/journal.pone.0145785 (PMC4689376; doi:10.1371/journal.pone.0145785)
Supplement: S4 Table — The chromosomal position of each CsLEA was mapped according to the C. sinensis Annotation Project (CAP). (DOCX) [file pone.0145785.s004.docx]

**S4 Table. Chromosomal locations of *CsLEAs*.** The chromosomal position of each *CsLEA* was mapped according to the *C. sinensis* Annotation Project (CAP).

| **Gene name** | **Chromosomal region** |
| --- | --- |
| *CsLEA1* | chr7:21,304,909..21,305,656 |
| *CsLEA2* | chr7:21,304,909..21,305,656 |
| *CsLEA3* | chr1:1,926,953..1,927,407 |
| *CsLEA4* | chrUn:34,061,402..34,062,336 |
| *CsLEA5* | chrUn:5,591,713..5,592,374 |
| *CsLEA6* | chr5:1,417,466..1,418,140 |
| *CsLEA7* | chr8:362,515..366,652 |
| *CsLEA8* | chrUn:6,738,433..6,740,247 |
| *CsLEA9* | chr3:25,675,425..25,677,293 |
| *CsLEA10* | chr1:21,169,830..21,171,339 |
| *CsLEA11* | chr1:25,230,952..25,232,699 |
| *CsLEA12* | chr5:1,417,466..1,418,140 |
| *CsLEA13* | chr5:881,089..881,718 |
| *CsLEA14* | chr2:18,822,408..18,822,971 |
| *CsLEA15* | chr2:4,495,832..4,496,734 |
| *CsLEA16* | chr2:4,495,832..4,496,734 |
| *CsLEA17* | chr2:4,493,360..4,494,339 |
| *CsLEA18* | chrUn:11,348,200..11,354,308 |
| *CsLEA19* | chr2:4,490,550..4,491,341 |
| *CsLEA20* | chr6:15,215,443..15,216,901 |
| *CsLEA21* | chr6:15,211,053..15,212,190 |
| *CsLEA22* | chr6:15,196,658..15,200,891 |
| *CsLEA23* | chr8:602,727..605,017 |
| *CsLEA24* | chr2:28,675,013..28,676,153 |
| *CsLEA25* | chr1:21,169,830..21,171,339 |
| *CsLEA26* | chr6:12,065,909..12,066,983 |
| *CsLEA27* | chr6:12,067,676..12,068,832 |
| *CsLEA28* | chr6:12,366,061..12,367,304 |
| *CsLEA29* | chr4:7,521,236..7,521,691 |
| *CsLEA30* | chr8:18,617,835..18,618,788 |
| *CsLEA31* | chr2:18,822,408..18,822,971 |
| *CsLEA32* | chr4:5,327,220..5,328,002 |
| *CsLEA33* | chr6:14,175,756..14,176,697 |
| *CsLEA34* | chr8:17,717,241..17,718,170 |
| *CsLEA35* | chr1:481,267..482,103 |
| *CsLEA36* | chr5:16,810,649..16,811,374 |
| *CsLEA37* | chr1:9,682,667..9,683,434 |
| *CsLEA38* | chrUn:19,184,629..19,189,061 |
| *CsLEA39* | chr4:7,927,708..7,928,758 |
| *CsLEA40* | chr1:17,692,227..17,694,612 |
| *CsLEA41* | chr4:15,054,762..15,057,168 |
| *CsLEA42* | chr5:21,885,960..21,889,423 |
| *CsLEA43* | chr4:15,415,461..15,417,320 |
| *CsLEA44* | chrUn:55,206,662..55,208,282 |
| *CsLEA45* | chrUn:11,348,200..11,354,308 |
| *CsLEA46* | chr6:14,198,888..14,199,577 |
| *CsLEA47* | chr5:1,422,047..1,423,139 |
| *CsLEA48* | chr5:34,350,070..34,351,232 |
| *CsLEA49* | chr9:2,313,276..2,314,373 |
| *CsLEA50* | chr1:19,935,258..19,935,656 |
| *CsLEA51* | chr1:19,937,398..19,937,821 |
| *CsLEA52* | chr8:2,369,539..2,370,513 |
| *CsLEA53* | chrUn:16,762,045..16,763,221 |
| *CsLEA54* | chr2:3,178,573..3,179,542 |
| *CsLEA55* | chr2:12,467,074..12,469,250 |
| *CsLEA56* | chr3:25,382,608..25,384,541 |
| *CsLEA57* | chr6:16,798,746..16,800,940 |
| *CsLEA58* | chr1:16,282,927..16,290,597 |
| *CsLEA59* | chr6:16,798,746..16,800,940 |
| *CsLEA60* | chr6:10,680,561..10,681,191 |
| *CsLEA61* | chr6:10,680,561..10,681,191 |
| *CsLEA62* | chr7:30,820,126..30,821,200 |
| *CsLEA63* | chr3:28,082,488..28,084,777 |
| *CsLEA64* | chr8:5,403,059..5,404,791 |
| *CsLEA65* | chr3:26,674,936..26,675,538 |
| *CsLEA66* | chr8:605,311..605,933 |
| *CsLEA67* | chr1:26,906,428..26,907,831 |
| *CsLEA68* | chr1:26,906,428..26,907,831 |
| *CsLEA69* | chr5:32,812,016..32,813,299 |
| *CsLEA70* | chr9:4,386,708..4,388,136 |
| *CsLEA71* | chr9:4,377,764..4,379,522 |
| *CsLEA72* | chr3:15,896,494..15,899,053 |
