# Supplementary material for: Late Embryogenesis Abundant (LEA) Constitutes a Large and Diverse Family of Proteins Involved in Development and Abiotic Stress Responses in Sweet Orange (Citrus sinensis L. Osb.)
Source: PLoS One. 2015 Dec 23;10(12):e0145785. doi: 10.1371/journal.pone.0145785 (PMC4689376; doi:10.1371/journal.pone.0145785)
Supplement: S5 Table — (DOCX) [file pone.0145785.s005.docx]

**S5 Table. Number of *cis*-elements ABRE (ACGTG) in the promoter regions of the selected *CsLEAs* for gene expression analysis.**

| **Group** | **Locus** | **Gene** | **N° of ABRE elements** |
| --- | --- | --- | --- |
| LEA_1 | orange1.1g031500 | *CsLEA4* | 18 |
| LEA_2 | orange1.1g047795 | *CsLEA11* | 14 |
| LEA_4 | orange1.1g036890 | *CsLEA53* | 11 |
| LEA_4 | orange1.1g035996 | *CsLEA54* | 10 |
| LEA_4 | orange1.1g009018 | *CsLEA55* | 11 |
| LEA_4 | orange1.1g037813 | *CsLEA56* | 14 |
| LEA_4 | orange1.1g041124 | *CsLEA57* | 20 |
| LEA_4 | orange1.1g045955 | *CsLEA58* | 13 |
| LEA_4 | orange1.1g048372 | *CsLEA59* | 17 |
| LEA_5 | orange1.1g040090 | *CsLEA60* | 16 |
| LEA_5 | orange1.1g042449 | *CSLEA61* | 13 |
| LEA_5 | orange1.1g045941 | *CsLEA62* | 20 |
| DEHYDRIN | orange1.1g026736 | *CsLEA63* | 25 |
| DEHYDRIN | orange1.1g042612 | *CsLEA67* | 11 |
| SMP | orange1.1g027886 | *CsLEA69* | 13 |
| SMP | orange1.1g038380 | *CsLEA71* | 16 |
| SMP | orange1.1g046001 | *CsLEA72* | 12 |
